# Behavior and weight correlates of weight-control efforts in Australian women living in disadvantage: The READI study

**DOI:** 10.1186/1479-5868-10-52

**Published:** 2013-04-26

**Authors:** Robert W Jeffery, Gavin Abbott, Kylie Ball, David Crawford

**Affiliations:** 1 Division of Epidemiology and Community Health, School of Public Health, University of Minnesota, Minnesota, USA; 2 Centre for Physical Activity and Nutrition Research, Deakin University, Melbourne, Australia

**Keywords:** Intentional, Weight-control, Food, Activity, Choice, Weight, Change

## Abstract

**Background:**

With increasing obesity rates worldwide, more and more people are actively attempting to lose weight or avoid weight gain, but relatively little is known about what specific behaviors comprise these efforts and which, if any, are associated with better weight control over time.

**Methods:**

This paper reports relationships between body weight, weight-control efforts and related behaviors over a three-year period in 1,634 Australian women. The women were purposefully recruited from 80 disadvantaged neighborhoods in Victoria, Australia. Weight loss efforts were categorized as trying to lose weight, trying to prevent weight gain and no weight-control efforts. Behavioral correlates examined included different kinds of physical activity and consumption of a number of specific foods types.

**Results and discussion:**

Self-reported body weight at baseline was higher in women trying to lose weight. Frequency of consumption of low energy density foods was positively associated with reported weight-control efforts, as was frequency of reported total and leisure-time physical activity. Longitudinal associations between changes in weight-control efforts and changes in behaviors were consistent with the cross-sectional findings. At three-year follow up, however, weight-control efforts were not associated with change in body weight. More detailed analyses of specific food choices suggested that part of the explanation of no effect of reported weight-control efforts and weight over time might be that people are not as well-informed as they should be about the energy density of some common foods. In particular, those reporting engagement in weight-control efforts reported reducing consumption of carbohydrate-containing foods such as bread and potatoes more than is justified by their energy content, while they reported increasing consumption of some high energy density foods (e.g., cheese and nuts).

**Conclusion:**

It is tentatively concluded that women living in disadvantaged neighborhoods understand messages about weight-control (more activity and foods with lower fat and lower energy density) but that some foods eaten more by women engaged in weight control may reduce the effectiveness of these efforts.

## Background

Gradual weight gain with age is normative in humans [1]. On average, weight increases steadily from late childhood through most of adulthood, as does the percentage of body weight that is fat. Unfortunately, the rate of this weight gain has increased dramatically in recent decades, which has resulted in equally dramatic increases in average body weight at all ages and we now face an epidemic of obesity [2,3]. Because excess weight is associated with a number of adverse health consequences [4], public health messages have increasingly encouraged the general population to change their eating and activity behaviors in order to lose weight, or to at least slow down weight gain [5].

People have listened to this advice to the extent that a substantial proportion of the population (especially those who are overweight or obese) are now concerned about their weight and report that they are trying to do something about it [6,7]. However, both weight loss and prevention have proved to be quite difficult for most. A substantial body of observational research over the last two decades has compared the behaviors of people trying to lose weight with those not trying to lose weight to explore why those attempting weight control are not more successful. Broadly speaking, behavioral correlates of absolute weight and of weight change have been consistent [8-17]. Foods with high energy density, i.e., fried foods, sugar-sweetened beverages, processed meats and high-fat desserts, are eaten more by heavier people and by those not trying to control their weight. Low-energy density foods, i.e., fruit and vegetables, are eaten more by those who are leaner and by those trying to control their weight. Similarly, higher energy expenditure in physical activity is associated with lower weight and with weight-control efforts, while sedentary behaviors are associated with higher body weight and with not trying to control weight.

Changes in food consumption, physical activity, and weight over time generally confirm that these patterns hold longitudinally within, as well as between, individuals. Whether intentional weight-loss efforts are helpful in weight control, however, is less clear. In teens and young adults, those who report dieting to lose weight are at increased risk of weight gain compared to those who do not [18]. The correlations between “healthy lifestyle” behaviors and weight are also less strong in populations of low socioeconomic status [10]. In the general adult population, however, reported weight-loss efforts tend to be associated with loss, or a reduced rate of weight gain, and several specific weight-control practices have been specifically associated with success, e.g., regular monitoring of body weight, regular physical activity, and preplanning meals. One of the most comprehensive assessments of weight-control strategies and weight was reported in a paper published by French et al. [7] in which middle-aged men and women were asked at annual intervals for four years the extent to which they had used each of 26 weight-control strategies in the previous year. The results strongly suggested that the vast majority of weight-control strategies reported were healthy, that strategies used for weight-gain prevention did not differ qualitatively from those that were used for weight loss, and that over time the more frequently individuals reporting using these weight-control strategies, the better they were able to control their weight [7].

Left unanswered in the above research is why, with increasing reports of weight-control efforts in the general population, we are not seeing a greater slowing down or reversal of the obesity epidemic. This paper describes an analysis of the cross-sectional and longitudinal relationships between self-reports of intentional weight-control efforts and physical activity behavior, sedentary behavior, intake of a number of specific food items, and body weight in a cohort of Australian women over a period of three years. The analyses focused on whether the specific food and activity choices associated with self-reported weight-control activities are achieving those goals. This paper adds to the current body of literature by filling in gaps in the existing literature regarding low SES populations as well as examining specific food choices in weight control in more detail.

## Methods

### Sample

This research project reports on data analysis using baseline and three-year follow-up data provided by women aged 18 to 46 years who were participants in the Resilience for Eating and Activity Despite Inequality (READI) study [19]. The age range was selected to focus on women of child bearing age, a group that is at high risk for weight gain. The study is following a cohort of women and children living in socioeconomically disadvantaged neighborhoods. The data presented here are from self-report measures provided by the women. Ethical approval for the study was granted by the Deakin University Human Research Ethics Committee, the Victorian Department of Education, and the Catholic Education Office.

Participants for READI were randomly selected using the electoral rolls from 40 rural and 40 urban suburbs (neighborhoods) within 200 km of Melbourne in the state of Victoria, Australia. As voting is compulsory for Australian adults, electoral rolls provide a relatively complete record of all Australian residents aged 18 years and over. Neighborhoods were randomly selected from the most socioeconomically disadvantaged third of all suburbs across Victoria, according to the Australian Bureau of Statistics’ (ABS) Socioeconomic Index for Areas [20].

A baseline survey was mailed to an initial sample of 11,940 women (150 women from each of the 80 neighborhoods or, where there were fewer than 150 women living in the neighborhood, all women within the age range within that neighborhood). The survey was distributed between August 2007 and January 2008. It was entirely self-report and assessed the women’s physical activity, eating behaviors, and a broad range of factors thought to influence these behaviors and obesity risk. Also included in the package were an invitation letter, a consent form, a $1 lottery ticket, and a teabag. A reminder protocol [21] was employed whereby letters were sent to nonresponders 10 days after the initial survey package was mailed. This was followed by a second reminder letter including another copy of the survey 10 days later. The surveys were initially pilot-tested with a convenience sample of 32 women aged 18 to 46 years and minor modifications were made for clarity based on the feedback received.

A total of 4,934 women returned a completed survey. Excluding from the denominator those whose surveys were marked ‘return to sender’ (n = 861) or who were otherwise ineligible (e.g., were deceased or were incorrectly denoted females on the electoral roll), this represented a response rate of 45%. The response rate was slightly higher in rural than in urban areas [22]. Of these 4,934, 571 were additionally excluded because they no longer lived in a READI suburb, nine were excluded because they were not within the desired age range (18 to 46 years), three were excluded because the survey was not completed by the woman it was addressed to, and two subsequently requested to be withdrawn from the study.

Three years following the baseline survey all respondents to the original survey who consented to further follow-up and remained eligible (n = 2,850) were sent a second survey, which repeated most of the questions in the baseline survey. One thousand nine hundred twelve (1,912) follow-up surveys were returned. The final sample for analysis excluded 278 additional women due to missing critical data (n = 107), pregnancy (n = 153), or reporting that that they were trying to gain weight (n = 31), leaving an analysis sample of 1,634.

### Measures

#### Sociodemographic characteristics

Participants were asked to provide sociodemographic information including their age, level of education attained, marital status, household income, employment status, country of birth, and number of dependent children. Education was categorized as low (No formal qualifications/Year 10 or equivalent), medium (Year 12 or equivalent/Trade or apprenticeship/Certificate or diploma), or high (University degree/Higher university degree). Marital status was categorized as married (Married/De facto), previously married (Separated/Divorced/Widowed), or never married. Household income was categorized as low ($0 to $699/week), medium ($700 to $1499/week), high ($1500+/week), or undisclosed (including participants who either refused to disclose their income or did not know how much). Employment status was categorized as working full-time, working part-time, or not currently employed in paid work (Unemployed or laid off/Keeping house or raising children full-time/Studying full-time/Retired). Country of birth was categorized as Australia or Other. Finally, the categories for number of children were none, one, two, or three or more.

*Weight and body mass index (BMI).* Participants provided their height at baseline (T1) and their weight at baseline and follow-up (T2). Height and weight values were converted into metric (m/kg) values as necessary. Each participant’s change in weight from T1 to T2 was calculated by subtracting their T1 weight from their T2 weight. BMI was calculated (kg/m^2^) for each participant at baseline and follow-up.

#### Physical activity and sedentary behaviors

Physical activity was assessed at T1 and T2 using the long version of the International Physical Activity Questionnaire (IPAQ-L). The IPAQ-L is a self-administered survey with demonstrated test-retest reliability and validity [23]. Women reported the number of hours and minutes that they had engaged in job-related, transport, domestic (household/yard), and leisure-time physical activity over the last seven days. A global physical activity measure was subsequently produced by summing the amount of time for each of these four domains. The IPAQ-L was also used to measure the total amount of time spent sitting in the past week. Further items were included to assess specific sedentary activities undertaken while sitting. Women were asked how much time they had spent sitting watching TV and at a computer during the last seven days on both weekdays and weekend days. Total weekly minutes spent sitting watching TV and at a computer were calculated by combining the weekday and weekend minutes proportionately (i.e., multiplying the average weekday by 5 and the average weekend day by 2 and summing these scores). Finally, the amount of weekly screen time was produced by summing the minutes of TV and computer time. For each of the T1 physical activity and sedentary behavior measures, tertile splits were used to categorize women as spending a low, medium, or high amount of time engaged in the activity. In order to maintain consistency between time points these same tertile cutoffs were used to categorize behaviors at T2.

#### Dietary intake

At both T1 and T2 participants completed a Food Frequency Questionnaire (FFQ) that was based on several previously published and validated Australian surveys [24-26]. The FFQ was used to assess past month consumption frequency of the following foods and beverages: vegetables, fried potatoes, nonfried potatoes, fruit, bread, salty snacks, confectionery, cake/sweet biscuits, meat pies/sausage rolls, fast foods, pizza, red meat, meat products, chicken, fish, legumes, eggs, nuts, cheese, yogurt, pasta/rice/noodles, breakfast cereal, water, soft drink, diet soft drink, fruit juice, and alcohol. Response options for vegetable and fruit intake ranged from “I don’t eat vegetables/fruit” to “6 servings or more/day.” Response options for fried and nonfried potato intake ranged from “I don’t eat fried/nonfried potatoes” to “6 servings or more/week.” Response options for bread intake ranged from “I don’t eat bread” to “8 slices or more/day.” Response options ranged from “Never or less than once/month” to “6 or more times/day” for salty snacks, confectionery, cake/sweet biscuits, meat pies/sausage rolls, fast foods, pizza, red meat, meat products, chicken, fish, legumes, eggs, nuts, cheese, yogurt, pasta/rice/noodles, and breakfast cereal. Response options for water, soft drink, diet soft drink, and fruit juice intake ranged from “I don’t drink [this item]” to “10 or more serves/day.” Response options for alcohol intake ranged from “I don’t drink alcohol” to “10 or more glasses/day.”

For each of the T1 dietary intake measures, tertile splits were used to categorize women as having low, medium, or high intake of each food or drink item, and these same tertile cutoffs were used to categorize dietary intakes at T2.

#### Weight-control efforts

Women’s baseline weight-control intention was assessed by asking participants at T1, “Which of the following best describes you at the moment?” Response options were: (1) I am actively doing things to gain weight at the moment; (2) I am actively doing things to try to avoid gaining weight at the moment; (3) I am actively doing things to try to lose weight at the moment; or (4) I am not doing anything in particular for my weight at the moment. The wording of these questions were unique to this study and have not been independently “validated”. It is noted, however, that very similar questions have been found to be correlated with weight, weight related behaviors and change of both over time in other studies [7]. Only a small proportion of women (< 2%) reported doing anything to gain weight and these women were subsequently excluded from analyses. Based on their responses, the remaining women were allocated to three weight-control effort groups: (1) no weight-control effort; (2) trying to avoid gaining weight; and (3) trying to lose weight.

### Statistical analyses

Some outcomes used in the analysis were continuous and some were categorical. Because preliminary examination revealed that many of the continuous variables were highly skewed (e.g., large numbers of zeros) and could not be remedied by transformations, all outcomes were expressed categorically. Tertiles were used to maintain large enough group sized to permit meaningful analysis.

Initially, the three weight-control effort groups were compared in terms of their sociodemographic characteristics. A one-way ANOVA with Bonferroni post-hoc tests was used to examine group differences on continuous age, and chi-square tests for independence were used to analyze categorical measures. In order to look at cross-sectional associations between weight-control efforts and health behavior, a series of multinomial logistic regression (MLR) analyses were conducted. A model was analyzed for each of the physical activity, sedentary behavior, or dietary behavior outcome measures. T1 weight-control effort was a predictor.

Longitudinal associations between weight-control efforts and behavior were similarly examined with separate models including each of the T2 (Year 3) health behavior measures as outcomes, and T1 (Baseline) weight-control efforts as a predictor. Each longitudinal model also controlled for the corresponding T1 behavior measure, e.g., in the model with T2 fruit intake as the outcome, T1 fruit intake was included as a covariate. Additionally, linear regression analyses were conducted to examine associations between T1 weight-control effort and T1 and T2 weight and BMI. Longitudinal associations between T1 weight-control effort and weight and BMI change from T1 to T2 were also examined, and included either T1 weight or T1 BMI as a covariate. In each regression analysis, ‘no weight-control effort’ was the reference category for weight-control effort, and ‘low’ was the reference category for health behavior. All regression analyses controlled for education level, age, marital status, country of birth, and clustering by suburb, all assessed at T1, while the behavioral outcome models additionally controlled for T1 BMI. Statistical analysis was conducted using STATA 12.0. Statistical significance was set at p < .05 (two-tailed) for all analyses.

## Results

### Socio-demographic characteristics

Baseline sociodemographic characteristics for the three weight-control intention groups are shown in Table 1. The groups did not differ with respect to household income, employment status, or the number of children living with them. However, the groups did differ in terms of age, BMI, education, marital status, and country of birth. Women actively trying to avoid gaining weight were significantly older than those actively trying to lose weight. In addition, women actively trying to avoid gaining weight were more likely to have a higher level of education than the other two groups, and women actively trying to lose weight were less likely to have been born overseas.

**Table 1:** T1 (baseline) socio-demographic characteristics of sample according to weight-control intention categories

**T1 characteristics of women**	**Whole sample**		**T1 weight-control effort**						
	**(n = 1,634)**		**Not doing anything in particular**		**Actively trying to avoid gaining**		**Actively trying to lose weight**		**p**
			**(n = 507)**		**(n = 488)**		**(n = 639)**		
	n	(%)	n	(%)	n	(%)	n	(%)	
Age (Mean [SD])	36.5	(7.6)	36.5	(7.6)	37.4	(7.2)	35.8	(7.9)	**.**002
Education									**.**008
Low (did not complete high school)	364	(22.3)	131	(25.8)	99	(20.3)	134	(21.0)	
Medium (completed high school or equivalent)	799	(48.9)	244	(48.1)	222	(45.5)	333	(52.1)	
High (completed tertiary education)	471	(28.8)	132	(26.0)	**167**	**(32.2)**	172	(26.9)	
Marital status									.029
Married/de facto	1160	(71.0)	356	(70.2)	354	(72.7)	450	(70.4)	
Separated/divorced/widowed	137	(8.4)	54	(10.7)	43	(8.8)	40	(6.3)	
Never married	336	(20.6)	97	(19.1)	90	(18.5)	149	(23.3)	
Household income									.288
Low ($0-$699/week)	386	(23.6)	128	(25.2)	113	(23.2)	145	(22.7)	
Medium ($700-$1499/week)	643	(39.4)	213	(42.0)	182	(37.3)	248	(38.8)	
High ($1500+/week)	337	(20.6)	86	(17.0)	110	(22.5)	141	(22.1)	
Not disclosed	268	(16.4)	80	(15.8)	83	(17.0)	105	(16.4)	
Employment status									.549
Working full-time	589	(36.5)	172	(34.5)	177	(36.6)	240	(38.0)	
Working part-time	554	(34.3)	168	(33.7)	173	(35.8)	213	(33.7)	
Currently not employed in paid work	470	(29.1)	158	(31.7)	133	(27.5)	179	(28.3)	
Country of birth									.013
Australia	1508	(92.3)	462	(91.1)	441	(90.4)	605	(94.7)	
Other	126	(7.7)	45	(8.9)	47	(9.6)	**34**	**(5.3)**	
Number of dependent children:									.080
None	559	(34.5)	165	(32.9)	152	(31.4)	242	(38.2)	
One	285	(17.6)	90	(18.0)	81	(16.7)	114	(18.0)	
Two	465	(28.7)	142	(28.3)	161	(33.3)	162	(25.6)	
Three or more	309	(19.1)	104	(20.8)	90	(18.6)	115	(18.2)	

Note: Some column totals are lower than expected due to missing data. Bolded cells are significantly under-or over-represented.

### Weight-control effort and physical activity

Cross-sectional associations were found between weight-control intention and transport and leisure-time physical activity (see Table 2). Compared to women with no weight-control intention, women actively trying to lose weight were more likely to have engaged in a high amount of transport physical activity at T1. Compared to women with no weight-control intention, women actively trying to avoid gaining weight were more likely to have engaged in a medium or high amount of leisure-time physical activity. Similarly, women actively trying to lose weight were more likely to have engaged in a medium or high amount of leisure-time physical activity at T1.

**Table 2:** MLR analyses of cross-sectional associations between T1 weight-control effort and T1 behaviors

**T1 behavioral outcomes**^**a**^		**T1 weight-control effort**^**b**^					
		**Actively trying to avoid gaining weight**			**Actively trying to lose weight**		
		**OR**	**(95% CI)**	**p**	**OR**	**(95% CI)**	**p**
PA/SB:							
Global PA	Low (0–690 mins/week)						
	Medium (691–1960 mins/week)	1.15	(0.81, 1.63)	.435	1.23	(0.89, 1.70)	.203
	High (1961+ mins/week)	1.21	(0.88, 1.68)	.240	1.10	(0.78, 1.53)	.592
Work PA	Low (0 mins/week)						
	Medium (1–420 mins/week)	1.00	(0.68, 1.46)	.982	0.77	(0.55, 1.07)	.113
	High (421+ mins/week	0.95	(0.69, 1.32)	.765	0.71	(0.50, 1.02)	.064
Transport PA	Low (0–30 mins/week)						
	Medium (31–140 mins/week)	1.27	(0.88, 1.82)	.206	1.00	(0.72, 1.38)	.977
	High (141+ mins/week)	1.19	(0.89, 1.61)	.230	**1.51**	**(1.09, 2.11)**	**.014**
Domestic PA	Low (0–230 mins/week)						
	Medium (231–780 mins/week)	1.20	(0.87, 1.65)	.260	1.09	(0.80, 1.48)	.587
	High (781+ mins/week)	1.34	(0.97, 1.87)	.079	1.18	(0.85, 1.65)	.322
Leisure-time PA	Low (0–52 mins/week)						
	Medium (53–225 mins/week)	**1.81**	**(1.31, 2.50)**	**<.0005**	**2.06**	**(1.47, 2.88)**	**<.0005**
	High (226+ mins/week)	**3.97**	**(2.72, 5.79)**	**<.0005**	**5.71**	**(3.87, 8.45)**	**<.0005**
Sitting	Low (0–1770 mins/week)						
	Medium (1771–3115 mins/week)	0.87	(0.64, 1.19)	.379	0.81	(0.58, 1.14)	.239
	High (3116+ mins/week)	1.08	(0.80, 1.46)	.605	0.97	(0.70, 1.32)	.828
TV	Low (0–780 mins/week)						
	Medium (781–1320 mins/week)	0.93	(0.67, 1.28)	.642	1.20	(0.86, 1.68)	.274
	High (1321+ mins/week)	0.85	(0.63, 1.15)	.283	0.92	(0.68, 1.25)	.587
Computer	Low (0–270 mins/week)						
	Medium (271–1260 mins/week)	0.88	(0.63, 1.23)	.469	0.84	(0.65, 1.10)	.207
	High (1261+ mins/week)	1.10	(0.78, 1.53)	.592	1.03	(0.74, 1.42)	.878
Screen time	Low (0–1365 mins/week)						
	Medium (1366–2570 mins/week)	0.98	(0.68, 1.41)	.915	1.08	(0.78, 1.49)	.651
	High (2571+ mins/week)	0.98	(0.73, 1.31)	.888	1.02	(0.73, 1.41)	.914
Dietary Intake:							
Vegetables	Low (0–1 serves/day)						
	Medium (2 serves/day)	1.13	(0.80, 1.62)	.483	1.31	(0.88, 1.94)	.182
	High (3+ serves/day)	1.35	(0.96, 1.89)	.088	**1.44**	**(1.03, 2.02)**	**.035**
Potato (fried)	Low (<1 serve/week)						
	Medium (1 serve/week)	**0.72**	**(0.57, 0.93)**	**.010**	**0.52**	**(0.40, 0.68)**	**<.0005**
	High (2+ serves/week)	**0.47**	**(0.36, 0.62)**	**<.0005**	**0.45**	**(0.33, 0.61)**	**<.0005**
Potato (nonfried)	Low (0–1 serves/week)						
	Medium (2–3 serves/week)	**0.73**	**(0.53, 0.99)**	**.044**	**0.47**	**(0.34, 0.64)**	**<.0005**
	High (4+ serves/week)	**0.63**	**(0.43, 0.92)**	**.017**	**0.47**	**(0.33, 0.65)**	**<.0005**
Fruit	Low (<1 serve/day)						
	Medium (1 serve/day)	**1.52**	**(1.13, 2.05)**	**.006**	**1.71**	**(1.28, 2.29)**	**<.0005**
	High (2+ serves/day)	**1.59**	**(1.13, 2.23)**	**.007**	**2.66**	**(1.97, 3.59)**	**<.0005**
Bread	Low (0–1 slices/day)						
	Medium (2 slices/day)	0.87	(0.62, 1.22)	.427	**0.64**	**(0.46, 0.88)**	**.006**
	High (3+ slices/day)	0.91	(0.64, 1.28)	.573	**0.59**	**(0.41, 0.83)**	**.002**
Salty snacks	Low (<once/month)						
	Medium (1–3 times/month)	0.93	(0.65, 1.35)	.712	0.95	(0.65, 1.39)	.785
	High (1+ times/week)	0.80	(0.56, 1.13)	.206	0.70	(0.49, 1.01)	.056
Confectionery	Low (0–3 times/month)						
	Medium (Once/week)	0.99	(0.69, 1.43)	.974	0.83	(0.60, 1.16)	.279
	High (2+ times/week)	0.78	(0.58, 1.07)	.124	**0.57**	**(0.44, 0.75)**	**<.0005**
Cake/Sweet biscuits	Low (0–3 times/month)						
	Medium (Once/week)	1.02	(0.74, 1.41)	.911	**0.67**	**(0.52, 0.89)**	**.005**
	High (2+ times/week)	0.85	(0.65, 1.13)	.268	**0.69**	**(0.53, 0.91)**	**.008**
Meat pies/ Sausage rolls	Low (<once/month)						
	Medium (1–3 times/month)	0.89	(0.66, 1.21)	.467	**0.63**	**(0.49, 0.81)**	**<.0005**
	High (1+ times/week)	**0.50**	**(0.33, 0.74)**	**.001**	**0.33**	**(0.23, 0.47)**	**<.0005**
Fast foods	Low (<once/month)						
	Medium (1–3 times/month)	**0.66**	**(0.49, 0.88)**	**.005**	**0.63**	**(0.46, 0.86)**	**.003**
	High (1+ times/week)	**0.44**	**(0.30, 0.62)**	**<.0005**	**0.43**	**(0.31, 0.58)**	**<.0005**
Pizza	Low (<once/month)						
	Medium (1–3 times/month)	0.96	(0.93, 1.26)	.757	**0.79**	**(0.62, 0.99)**	**.045**
	High (1+ times/week)	0.79	(0.42, 1.26)	.255	**0.64**	**(0.44, 0.93)**	**.018**
Red meat	Low (0–1 times/week)						
	Medium (2 times/week)	1.04	(0.75, 1.45)	.813	0.71	(0.51, 1.01)	.054
	High (3+ times/week)	0.99	(0.69, 1.42)	.973	0.77	(0.51, 1.17)	.222
Meat products	Low (<once/month)						
	Medium (1–3 times/month)	0.83	(0.60, 1.15)	.259	**0.64**	**(0.47, 0.88)**	**.006**
	High (1+ times/week)	**0.64**	**(0.47, 0.87)**	**.004**	**0.60**	**(0.44, 0.83)**	**.002**
Chicken	Low (0–3 times/month)						
	Medium (Once/week)	0.99	(0.71, 1.37)	.934	0.75	(0.52, 1.07)	.116
	High (2+ times/week)	0.88	(0.62, 1.26)	.486	0.94	(0.66, 1.33)	.714
Fish	Low (0–3 times/month)						
	Medium (Once/week)	**1.64**	**(1.24, 2.17)**	**.001**	**1.36**	**(1.05, 1.76)**	**.020**
	High (2+ times/week)	**1.86**	**(1.28, 2.71)**	**.001**	**2.21**	**(1.53, 3.18)**	**<.0005**
Legumes	Low (<once/month)						
	Medium (1–3 times/month)	1.15	(0.85, 1.55)	.363	1.06	(0.76, 1.47)	.739
	High (1+ times/week)	1.24	(0.95, 1.62)	.108	**1.47**	**(1.11, 1.96)**	**.007**
Eggs	Low (0–3 times/month)						
	Medium (Once/week)	**1.39**	**(1.04, 1.84)**	**.024**	1.20	(0.92, 1.56)	.171
	High (2+ times/week)	0.99	(0.69, 1.41)	.947	1.16	(0.84, 1.61)	.367
Nuts	Low (<once/month)						
	Medium (1–3 times/month)	1.18	(0.84, 1.67)	.333	1.10	(0.83, 1.48)	.504
	High (1+ times/week)	**1.68**	**(1.21, 2.33)**	**.002**	**1.56**	**(1.15, 2.12)**	**.005**
Cheese	Low (0–1 times/week)						
	Medium (2 times/week)	1.23	(0.91, 1.66)	.177	1.11	(0.82, 1.50)	.498
	High (3+ times/week)	**1.34**	**(1.00, 1.79)**	**.049**	1.01	(0.75, 1.38)	.926
Yogurt	Low (<once/month)						
	Medium (1–4 times/month)	1.36	(0.99, 1.86)	.059	1.19	(0.86, 1.64)	.301
	High (2+ times/week)	**1.95**	**(1.43, 2.66)**	**<.0005**	**2.22**	**(1.64, 2.99)**	**<.0005**
Pasta/Rice/ Noodles	Low (0–1 times/week)						
	Medium (2 times/week)	1.25	(0.92, 1.69)	.156	0.99	(0.75, 1.30)	.920
	High (3+ times/week)	1.06	(0.74, 1.53)	.752	0.79	(0.56, 1.13)	.203
Breakfast cereal	Low (0–3 times/month)						
	Medium (1–6 times/week)	**1.48**	**(1.07, 2.04)**	**.018**	**1.47**	**(1.12, 1.93)**	**.005**
	High (1+ times/day)	**1.58**	**(1.16, 2.14)**	**.003**	**1.38**	**(1.01, 1.88)**	**.044**
Water	Low (0–3 serves/day)						
	Medium (4–7 serves/day)	1.34	(0.99, 1.82)	.060	**1.70**	**(1.23, 2.36)**	**.001**
	High (8+ serves/day)	**1.89**	**(1.32, 2.70)**	**<.0005**	**3.22**	**(2.42, 4.30)**	**<.0005**
Soft drink	Low (0 serves/day)						
	Medium (<1 serves/day)	0.83	(0.64, 1.09)	.184	**0.63**	**(0.47, 0.84)**	**.002**
	High (1+ serves/day)	**0.51**	**(0.34, 0.75)**	**.001**	**0.30**	**(0.21, 0.43)**	**<.0005**
Diet soft drink	Low (0 serves/day)						
	Medium (<1 serves/day)	**1.82**	**(1.33, 2.48)**	**<.0005**	**2.33**	**(1.66, 3.26)**	**<.0005**
	High (1+ serves/day)	1.03	(0.68, 1.54)	.898	**1.63**	**(1.06, 2.48)**	**.024**
Fruit juice	Low (0 serves/day)						
	Medium (<1 serves/day)	0.89	(0.65, 1.21)	.452	0.89	(0.58, 1.08)	.139
	High (1+ serves/day)	0.85	(0.59, 1.24)	.400	**0.63**	**(0.45, 0.88)**	**.006**
Alcohol	Low (0–1 serves/day)						
	Medium (2–3 serves/day)	0.94	(0.74, 1.19)	.595	1.04	(0.80, 1.36)	.773
	High (4+ serves/day)	1.21	(0.88, 1.66)	.246	1.07	(0.79, 1.45)	.666

^a^ ‘Low’ is the reference category for all outcomes.

^b^ ‘Not doing anything in particular regarding weight’ is the reference category for weight-control intention.

Note: All analyses controlled for T1 BMI, maternal education, age, marital status, country of birth, and clustering by suburb. Bolded odds-ratios were significant.

As can be seen in Table 3, longitudinal associations were found between weight-control effort and global, transport, and leisure-time physical activity. Women actively trying to avoid gaining weight were more likely to have engaged in a high amount of global physical activity and leisure-time physical activity, compared to women not engaged in weight-control at all. Women actively trying to lose weight were more likely to have engaged in a high amount of global physical activity, transport physical activity, and leisure-time physical activity at T2, compared to women with no weight-control effort.

**Table 3:** Longitudinal associations between T1 weight-control intention and T2 behaviors, adjusting for T1 behavior

**T2 behavioral outcomes**^**a**^		**T1 weight-control effort**^**b**^					
		**Actively trying to avoid gaining weight**			**Actively trying to lose weight**		
		**OR**	**(95% CI)**	**p**	**OR**	**(95% CI)**	**p**
PA/SB:							
Global PA	Low (0–690 mins/week)						
	Medium (691–1960 mins/week)	1.13	(0.82, 1.55)	.453	1.20	(0.86, 1.67)	.281
	High (1961+ mins/week)	**1.54**	**(1.05, 2.27)**	**.029**	**1.97**	**(1.33, 2.93)**	**.001**
Work PA	Low (0 mins/week)						
	Medium (1–420 mins/week)	0.95	(0.65, 1.40)	.813	1.01	(0.68, 1.48)	.975
	High (421+ mins/week	1.17	(0.73, 1.87)	.520	1.17	(0.72, 1.88)	.533
Transport PA	Low (0–30 mins/week)						
	Medium (31–140 mins/week)	1.22	(0.82, 1.82)	.320	1.27	(0.91, 1.78)	.156
	High (141+ mins/week)	1.47	(0.98, 2.21)	.065	**1.55**	**(1.03, 2.32)**	.**036**
Domestic PA	Low (0–230 mins/week)						
	Medium (231–780 mins/week)	1.35	(0.60, 2.02)	.144	1.04	(0.72, 1.50)	.849
	High (781+ mins/week)	1.01	(0.68, 1.53)	.935	1.16	(0.77, 1.74)	.473
Leisure-time PA	Low (0–52 mins/week)						
	Medium (53–225 mins/week)	0.98	(0.67, 1.45)	.938	1.15	(0.76, 1.74)	.502
	High (226+ mins/week)	**1.87**	**(1.33, 2.63)**	**<.0005**	**2.44**	**(1.61, 3.70)**	**<.0005**
Sitting	Low (0–1770 mins/week)						
	Medium (1771–3115 mins/week)	0.81	(0.59, 1.13)	.224	0.93	(0.69, 1.24)	.609
	High (3116+ mins/week)	0.84	(0.58, 1.21)	.352	0.90	(0.59, 1.38)	.638
TV	Low (0–780 mins/week)						
	Medium (781–1320 mins/week)	**0.58**	**(0.39, 0.87)**	**.008**	0.72	(0.50, 1.05)	.090
	High (1321+ mins/week)	0.66	(0.44, 1.01)	.054	0.70	(0.47, 1.04)	.078
Computer	Low (0–270 mins/week)						
	Medium (271–1260 mins/week)	0.84	(0.62, 1.15)	.277	**0.71**	**(0.51, 1.00)**	**.048**
	High (1261+ mins/week)	0.73	(0.51, 1.05)	.093	0.94	(0.62, 1.43)	.775
Screen time	Low (0–1365 mins/week)						
	Medium (1366–2570 mins/week)	**0.61**	**(0.44, 0.87)**	**.006**	0.73	(0.51, 1.04)	.079
	High (2571+ mins/week)	**0.62**	**(0.43, 0.91)**	**.015**	0.91	(0.61, 1.35)	.636
Dietary Intake:							
Vegetables	Low (0–1 serves/day)						
	Medium (2 serves/day)	1.35	(0.93, 1.95)	.111	**1.56**	**(1.04, 2.33)**	**.030**
	High (3+ serves/day)	**1.40**	**(1.01, 1.95)**	**.046**	1.39	(0.95, 2.04)	.087
Potato (fried)	Low (<1 serve/week)						
	Medium (1 serve/week)	**0.68**	**(0.48, 0.97)**	**.035**	0.78	(0.55, 1.11)	.160
	High (2+ serves/week)	0.73	(0.48, 1.10)	.132	0.70	(0.49, 1.00)	.052
Potato (nonfried)	Low (0–1 serves/week)						
	Medium (2–3 serves/week)	0.88	(0.62, 1.26)	.494	0.81	(0.59, 1.09)	.165
	High (4+ serves/week)	0.84	(0.57, 1.22)	.358	0.80	(0.55, 1.17)	.251
Fruit	Low (<1 serve/day)						
	Medium (1 serve/day)	1.03	(0.72, 1.50)	.856	0.82	(0.57, 1.19)	.306
	High (2+ serves/day)	**1.57**	**(1.09, 2.25)**	**.015**	1.14	(0.79, 1.65)	.488
Bread	Low (0–1 slices/day)						
	Medium (2 slices/day)	0.82	(0.60, 1.10)	.184	0.84	(0.60, 1.16)	.280
	High (3+ slices/day)	**0.65**	**(0.47, 0.91)**	**.011**	0.72	(0.49, 1.06)	.096
Salty snacks	Low (<once/month)						
	Medium (1–3 times/month)	0.89	(0.63, 1.27)	.528	0.78	(0.56, 1.11)	.171
	High (1+ times/week)	0.76	(0.54, 1.06)	.107	0.69	(0.47, 1.01)	.056
Confectionery	Low (0–3 times/month)						
	Medium (Once/week)	0.94	(0.68, 1.29)	.701	1.08	(0.80, 1.45)	.610
	High (2+ times/week)	1.05	(0.76, 1.43)	.782	1.05	(0.73, 1.51)	.794
Cake/Sweet biscuits	Low (0–3 times/month)						
	Medium (Once/week)	1.16	(0.86, 1.57)	.324	1.21	(0.86, 1.68)	.274
	High (2+ times/week)	0.82	(0.59, 1.15)	.260	1.05	(0.76, 1.44)	.785
Meat pies/ Sausage rolls	Low (<once/month)						
	Medium (1–3 times/month)	**0.73**	**(0.54, 1.00)**	**.049**	**0.66**	**(0.48, 0.89)**	**.008**
	High (1+ times/week)	**0.56**	**(0.38, 0.85)**	**.006**	0.81	(0.50, 1.30)	.374
Fast foods	Low (<once/month)						
	Medium (1–3 times/month)	1.10	(0.83, 1.46)	.501	0.99	(0.73, 1.34)	.943
	High (1+ times/week)	0.99	(0.65, 1.53)	.979	0.95	(0.62, 1.48)	.837
Pizza	Low (<once/month)						
	Medium (1–3 times/month)	0.98	(0.76, 1.27)	.879	1.15	(0.93, 1.43)	.206
	High (1+ times/week)	0.86	(0.51, 1.43)	.558	0.94	(0.58, 1.52)	.805
Red meat	Low (0–1 times/week)						
	Medium (2 times/week)	1.04	(0.72, 1.49)	.837	1.20	(0.88, 1.65)	.250
	High (3+ times/week)	0.88	(0.58, 1.34)	.550	1.07	(0.77, 1.50)	.682
Meat products	Low (<once/month)						
	Medium (1–3 times/month)	0.87	(0.64, 1.18)	.355	0.89	(0.64, 1.25)	.514
	High (1+ times/week)	0.77	(0.61, 1.29)	.529	0.77	(0.54, 1.09)	.134
Chicken	Low (0–3 times/month)						
	Medium (Once/week)	0.83	(0.48, 1.42)	.492	0.78	(0.47, 1.31)	.347
	High (2+ times/week)	1.00	(0.62, 1.60)	.994	0.77	(0.49, 1.20)	.247
Fish	Low (0–3 times/month)						
	Medium (Once/week)	1.10	(0.83, 1.47)	.515	0.82	(0.61, 1.10)	.186
	High (2+ times/week)	**1.46**	**(1.05, 2.03)**	**.024**	**1.43**	**(1.04, 1.96)**	**.029**
Legumes	Low (<once/month)						
	Medium (1–3 times/month)	1.19	(0.80, 1.75)	.390	0.91	(0.69, 1.19)	.495
	High (1+ times/week)	**1.62**	**(1.19, 2.21)**	**.002**	1.35	(0.94, 1.93)	.108
Eggs	Low (0–3 times/month)						
	Medium (Once/week)	1.15	(0.77, 1.71)	.500	1.12	(0.76, 1.66)	.553
	High (2+ times/week)	**1.45**	**(1.01, 2.09)**	**.045**	**1.73**	**(1.17, 2.54)**	**.006**
Nuts	Low (<once/month)						
	Medium (1–3 times/month)	0.78	(0.53, 1.14)	.192	1.01	(0.74, 1.37)	.959
	High (1+ times/week)	1.04	(0.73, 1.50)	.823	1.16	(0.80, 1.67)	.437
Cheese	Low (0–1 times/week)						
	Medium (2 times/week)	1.10	(0.82, 1.47)	.531	0.90	(0.67, 1.20)	.470
	High (3+ times/week)	0.91	(0.65, 1.28)	.592	0.88	(0.62, 1.27)	.499
Yogurt	Low (<once/month)						
	Medium (1–4 times/month)	1.11	(0.74, 1.68)	.619	0.78	(0.54, 1.14)	.202
	High (2+ times/week)	1.21	(0.82, 1.78)	.345	0.94	(0.63, 1.38)	.737
Pasta/Rice/ Noodles	Low (0–1 times/week)						
	Medium (2 times/week)	0.78	(0.58, 1.05)	.106	**0.74**	**(0.57, 0.96)**	**.024**
	High (3+ times/week)	**0.49**	**(0.32, 0.76)**	**.001**	0.75	(0.50, 1.13)	.167
Breakfast cereal	Low (0–3 times/month)						
	Medium (1–6 times/week)	1.15	(0.85, 1.54)	.363	1.16	(0.84, 1.59)	.365
	High (1+ times/day)	**1.48**	**(1.02, 2.16)**	**.040**	**1.66**	**(1.15, 2.39)**	**.006**
Water	Low (0–3 serves/day)						
	Medium (4–7 serves/day)	**1.53**	**(1.08, 2.15)**	**.016**	0.91	(1.23, 2.36)	.540
	High (8+ serves/day)	**2.03**	**(1.46, 2.83)**	**<.0005**	1.31	(0.93, 1.84)	.117
Soft drink	Low (0 serves/day)						
	Medium (<1 serves/day)	0.81	(0.59, 1.11)	.191	0.97	(0.71, 1.32)	.824
	High (1+ serves/day)	0.90	(0.54, 1.50)	.688	0.74	(0.47, 1.17)	.201
Diet soft drink	Low (0 serves/day)						
	Medium (<1 serves/day)	0.93	(0.65, 1.33)	.680	1.30	(0.87, 1.95)	.203
	High (1+ serves/day)	0.72	(0.44, 1.20)	.217	1.18	(0.74, 1.90)	.480
Fruit juice	Low (0 serves/day)						
	Medium (<1 serves/day)	**0.49**	**(0.36, 0.66)**	**<.0005**	**0.48**	**(0.35, 0.67)**	**<.0005**
	High (1+ serves/day)	**0.59**	**(0.41, 0.83)**	**.003**	**0.50**	**(0.34, 0.72)**	**<.0005**
Alcohol	Low (0–1 serves/day)						
	Medium (2–3 serves/day)	1.10	(0.77, 1.56)	.596	1.23	(0.93, 1.64)	.150
	High (4+ serves/day)	1.28	(0.89, 1.84)	.190	1.20	(0.83, 1.73)	.340

^a^ ‘Low’ is the reference category for all outcomes.

^b^ ‘Not doing anything in particular regarding weight’ is the reference category for weight-control effort.

Note: All analyses controlled for T1 behavior, BMI, maternal education, age, marital status, country of birth, and clustering by suburb. Bolded odds-ratios were significant.

### Weight-control effort and sedentary behavior

None of the sedentary behavior measures were cross-sectionally associated with weight-control effort (see Table 2). However, longitudinal associations were found between weight-control efforts and TV, computer, and total screen time (see Table 3). Women actively trying to avoid gaining weight were less likely to have spent a medium amount of time watching TV, and less likely to have engaged in a medium or high amount of screen time at T2, compared to women with no weight-control intention. Women actively trying to lose weight were more likely to have engaged in a medium amount of computer time at T2, compared to women with no weight-control effort.

### Weight-control effort and dietary behavior

As can be seen in Table 2, cross-sectional associations were found between weight-control intention and a number of dietary behaviors. For example, women actively trying to avoid gaining weight were less likely to have a high consumption frequency of fried potatoes, non-fried potatoes, meat pies/sausage rolls, fast foods, meat products, and soft drinks at T1, compared to women engaged in no weight-control effort. Conversely, women actively trying to avoid gaining weight were more likely to have a high consumption frequency of fruit, fish, nuts, cheese, yogurt, breakfast cereal, and water at T1, compared to women with no weight-control effort.

Women actively trying to lose weight were less likely to have a high consumption frequency of fried potatoes, non-fried potatoes, bread, confectionery, cake/sweet biscuits, meat pies/sausage rolls, fast foods, pizza, meat products, soft drinks, and fruit juice at T1, compared to women with no weight-control effort. Women actively trying to lose weight were more likely to have a high consumption frequency of vegetables, fruit, fish, legumes, nuts, yogurt, breakfast cereal, water, and diet soft drinks at T1, compared to women with no weight-control effort.

A number of longitudinal associations were also found between weight-control effort and dietary behaviors (see Table 3). For example, women actively trying to avoid gaining weight were less likely to have a high consumption frequency of bread, meat pies/sausage rolls, pasta/rice/noodles, and fruit juice, and more likely to have a high consumption frequency of vegetables, fruit, fish, legumes, eggs, breakfast cereal, and water at T2, compared to women with no weight-control effort. Women actively trying to lose weight were less likely to have a high consumption frequency of fruit juice, and more likely to have a high consumption frequency of fish, eggs, and breakfast cereal at T2, compared to women with no weight-control effort.

### Weight-control effort, weight, and weight change

Weight and BMI characteristics for the three weight-control intention groups are shown in Table 4. Women actively trying to lose weight were significantly heavier and had higher BMI at both T1 and T2 than women with no weight-control effort or those actively trying to avoid gaining weight. All three weight-control groups had an increase in weight and BMI from T1 to T2. No longitudinal associations were found between weight-control efforts and change in weight or BMI.

**Table 4:** Weight and BMI of sample according to weight-control intention categories

	**T1 weight-control effort**						
	**Not doing anything in particular**		**Actively trying to avoid gaining**		**Actively trying to lose weight**		
	**M**	**(SD)**	**M**	**(SD)**	**M**	**(SD)**	**p**
T1 weight	67.3	(16.6)	65.9	(12.3)	77.6	(16.4)	<.0005
T2 weight	69.4	(17.9)	67.6	(13.0)	78.6	(17.3)	<.0005
Weight change	2.1	(7.1)	1.7	(5.2)	1.1	(7.7)	.280
T1 BMI	25.0	(6.2)	24.4	(4.3)	28.7	(6.0)	<.0005
T2 BMI	25.8	(6.7)	25.0	(4.6)	29.0	(6.3)	<.0005
BMI change	0.8	(2.6)	0.6	(1.9)	0.4	(2.8)	.207

Note: All analyses controlled for maternal education, age, marital status, country of birth, and clustering by suburb. The models for BMI and weight change included T1 BMI and T1 weight, respectively, as additional covariates.

## Discussion

This study sought to examine whether specific food and activity choices are associated with weight-control activities and with weight change. The findings of the present study are generally consistent with those of similar studies in other populations regarding weight-control efforts, weight and behaviors. Leaner women report eating diets that are lower in foods of high energy density and higher in foods with low energy density, and also report more physical activity [15]. Cross-sectional correlates of reported efforts at weight-control and behaviors are similar to those for the same behaviors and body weight and longitudinal findings for behaviors and weight-control efforts are also consistent with these patterns [12,13]. Increases in consumption of low energy dense foods and of physical activity are reported in those trying to control their weight compared to those who are not, while decreases are reported in consumption of foods with high energy density and to a lesser degree in sedentary behaviors. In addition, similar to the report of French et al. [7], there seemed to be no noticeable qualitative difference between the behavioral correlates of effort to lose weight as efforts to avoid weight gain, although effect sizes are generally larger for efforts to lose weight than they are for efforts to avoid weight gain, perhaps indicating greater intensity of effort in the weight losers.

One difference between the present findings and previous work in this area, however, was that we found no relationships between change in weight over time and reported efforts to lose weight or avoid gaining weight. Our sample was smaller than that of some previous studies, so that lack of power is a plausible contributing explanation [27]. Both the weight gain prevention group and the no weight-control effort group were also relatively lean with mean BMI at baseline within the normal range. However, our sample was also purposefully selected to over-represent lower SES women by drawing a greater sample from socioeconomically disadvantaged neighborhoods, and relationships seen previously in other populations have been more ambiguous in lower SES groups [10].

Considering the overall question of why weight-control effort in the general population are not more successful, two logical possibilities are that people know what to do but implement this knowledge with insufficient frequency or intensity and that their knowledge is not complete enough or specific enough to avoid errors in behavioral choices that undermine their efforts. Conceptually these factors might be particularly important in low SES populations [28]. Although we have little direct evidence to bring to bear on intensity of efforts, in the present results we note five observations that could be interpreted as supporting an incomplete knowledge hypothesis. There were two food items in particular that women trying to lose weight reported eating less of that are not especially high in energy density, namely regular potatoes and bread. While avoiding these foods has been recommended in low carbohydrate diets, that at various points in time have been quite popular, making concerted efforts to reduce their intake may not be the best focal point from the perspective of energy balance. Similarly, we note two food items, nuts and cheese, whose increase in those trying to lose or maintain weight could be self-defeating, since nuts and cheese are high in energy density. The fifth choice that might be suspect was increased frequency of egg consumption in those trying to control their weight. Increased egg consumption is usually not recommended in public health messages about weight control, although it is noted that some recent empirical data has supported the idea that it might be helpful [29]. We are not aware of similar item specific data being reported in other studies, but suggest that further research on potentially counter-productive food choices among dieters is merited and might be informative for formulating weight-related nutrition messages. It should be noted, however, that the inference that the patterns of food selections seen in this population were mediated by knowledge and beliefs is not directly verifiable here because we did not have a measure of nutrition knowledge or beliefs. Food selections could have alternatively been the result of a number of factors unrelated to knowledge (e.g., lack of access or budgetary constraints). More direct confirmation would be necessary before making recommendations for public health messaging.

A second finding of the present study that merits discussion is the asymmetry of findings with respect to physical activity and sedentary behavior [30]. Our data show consistent positive relationships between total self-reported activity and its leisure-time and transport components and both body weight cross-sectionally and weight-control activity both cross-sectionally and longitudinally, but only very weak associates with sedentary behavior, e.g., sitting and screen time. At face value, the finding suggests that active behavior is more important than sedentary behavior for weight-control, however, it also points to possible methodological problems in measurement, e.g., frequency and duration of active behaviors may be better recalled than sedentary behaviors because they are more discrete. More attention to the methods for measuring activity components is recommended.

This study had a number of strengths. These include a unique population of women residing in neighborhoods of relative deprivation, a behavioral survey with broad assessment of obesity related behaviors in the domains of both energy intake and energy expenditure, and a lengthy follow-up period. Offsetting weaknesses include a modest response rate to the survey, which probably makes the sample less representative of the entire population of the areas from which they were drawn than is desirable, complete reliance on self-report measures, some of which were not validated, and measurement tools that were not fashioned specifically on the hypothesis being investigated (e.g., self-reports of food intake over the last three months may not be reflective of diet intake over the three years between observations). It is also noted that the large number of statistical test examined increases the likelihood of type two error. It is believed in sum, however, that the investigation was unique and that the findings advance understanding of what it means behaviorally when people say that they are trying to lose or maintain weight and how those activities might impact the time course of weight over a period of years.

The methodological limitations of this research limit strong recommendations for practitioners or public health messaging. However, should it prove to be the case that failure of self-initiated weight loss and weight gain prevention efforts is related to inadequate knowledge about the energy content of foods, modified public health messaging related to food choices may be in order.

## Competing interests

The authors declare that they have no competing interests.

## Authors’ contributions

Dr. RWJ conceptualized the paper, directed the analyses and wrote the paper. Dr. KB is leader of the READI project and thus, was responsible for collecting the data. She also participated in all aspects of the analysis and writing of the paper. Dr. GA was the principle designer of the analysis plan, but also assisted with literature review, wrote much of the analysis section and provided constructive feedback on other aspects of the paper. Dr. DC was particularly valuable in framing the research questions for the paper and contributed significantly to most other aspects as well. All authors read and approved the final manuscript.
